# The β1 adrenoceptor (AR) blocker metoprolol normalises βAR cascade and caveolar protein expression and restores β1AR responsiveness in failing right ventricular myocytes

**DOI:** 10.1016/j.jmccpl.2026.100857

**Published:** 2026-07-17

**Authors:** Ruth Norman, Rachel Stones, Amelia S. Power, Ed White, Sarah C. Calaghan, Ewan D. Fowler

**Affiliations:** a School of Biomedical Sciences, Faculty of Biological Sciences, University of Leeds, UK; b Department of Physiology, University of Auckland, Auckland, New Zealand; c School of Biosciences, College of Biological and Life Sciences, University of Cardiff, Cardiff, UK

**Keywords:** Pulmonary arterial hypertension, beta-adrenoceptor, Caveolae, beta-blocker, Heart failure, Right ventricle, Monocrotaline

## Abstract

Right ventricular (RV) failure is the leading cause of death in pulmonary artery hypertension (PAH) and treatments that preserve RV contractility are urgently required. β-adrenoceptor blockers (BB) are used clinically in left ventricular failure to improve β-adrenoceptor (AR) responsiveness but it is not known whether they confer the same benefits in RV failure. Here we tested this using the rat monocrotaline (MCT) model of PAH. When PAH was established, treatment commenced with β1AR-selective metoprolol (10 mg/kg/day; MCT + BB group) or vehicle (MCT group). In isolated RV myocytes from MCT, inotropic and lusitropic responses to β1AR stimulation were blunted vs. non-failing controls (CON), and this was recovered by BB treatment. Comparable effects on amplitude/kinetics of the Ca^2+^ transient were observed. The impact of RV failure and BB on β1AR responsiveness could be explained by altered expression of proteins of the βAR cascade and its regulatory domain, the caveola. Expression of β1AR, adenylyl cyclase 5/6, caveolin 1 & 3, and cavin 1 were decreased in RV from MCT, whereas G protein receptor kinase was increased. These changes were reversed by BB treatment, with the exception of caveolin 1. A computational model of cardiac myocyte β1AR signalling, incorporating observed changes in protein expression, showed that BB treatment recovered the β1AR-cAMP signals in caveolar and extra-caveolar compartments. Modelling indicated that recovery of adenylyl cyclase 5/6 was the main factor responsible for the beneficial impact of BB treatment on failing RV myocyte contractility. As RV function critically influences symptoms and mortality, this work supports the potential use of BB as a novel treatment for PAH-RV failure.

## Highlights

• There are few treatments available to improve RV function in pulmonary artery hypertension (PAH)
• β-adrenoceptor blockers (BB) are effective in left ventricular failure, but their effect on failing RV in PAH is unclear
• BB treatment of monocrotaline (MCT) rats improved RV myocyte contractility during β-adrenergic stimulation
• BB attenuated changes in β-adrenergic signalling cascade protein expression and subcellular distribution
• These data indicate BB may preserve β-adrenergic responsiveness in preclinical PAH with RV failure and improve function

## Graphical abstract

**Figure:**
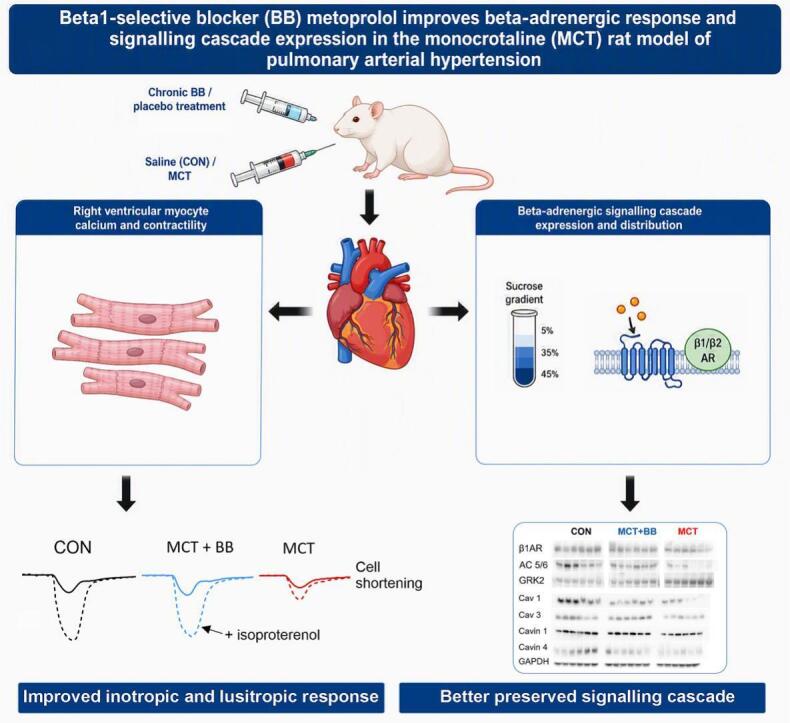

## 1 Introduction

Pulmonary arterial hypertension (PAH) affects relatively young individuals and carries a poor prognosis, with median survival around 3 years [1]. It occurs due to restricted flow through the pulmonary arterial circulation which increases pulmonary artery pressure. This places an additional load on the right ventricle (RV), resulting in RV failure [2]. Current treatments target remodelling of the pulmonary vasculature and increased pressure in the pulmonary circulation. However, it is the function of the RV that determines prognosis in PAH, and RV failure is the leading cause of death in these patients [3]. RV failure can progress even when pulmonary vascular resistance is controlled [4]. Despite this, at present no treatments for PAH directly target the failing RV [5]. New RV-directed approaches are urgently required to treat PAH.

In left ventricular (LV) heart failure, increased sympathetic drive initially compensates for impaired cardiac function by increasing contractility of the myocardium. However, this change in autonomic balance ultimately drives maladaptive changes which include desensitization of the β-adrenoceptor (AR) pathway and decreased cardiac contractility [6]. Blunted βAR responsiveness can be explained by reduced β1AR expression and receptor-G protein coupling, decreased adenylyl cyclase activity, and increased Gαi expression and G-protein receptor kinase activity [6]. Importantly, similar changes in βAR cascade component expression and activity have been reported in RV failure [7], [8]. Increased sympathetic activity in PAH patients is associated with functional deterioration and increased mortality [9].

βAR blockers (BB) are an effective treatment in LV failure. They restore βAR responsiveness, improve cardiac function and improve long-term survival [10]. However, recent guidelines do not recommend BB as a treatment for PAH and RV failure unless required to address comorbidities such as high blood pressure, dysrhythmia [11]. Concerns have been raised surrounding their use [12]. Nevertheless, recent small clinical trials have shown that BB treatment is well-tolerated in PAH patients and may confer some benefits including enhanced RV function [13], faster heart rate recovery after exercise [3] and a trend for increased 6 min walk distance [14]. A recent large multi-centre study found that patients with newly diagnosed PAH receiving BB for co-morbidities such as arrhythmias, coronary artery disease or hypertension, were not at increased risk of adverse outcomes compared to propensity score-matched control patients, whereas in patients without co-morbidities BB increased the risk [15]. Better understanding of the myocardial adaptations following chronic BB treatment could inform which PAH patients may stand to benefit, or where they should be avoided.

There is growing evidence from pre-clinical rodent models of PAH that BB improve myocardial excitation-contraction coupling and capillary density, and reduce fibrosis and inflammation [16], [17]. However, to date there is no direct demonstration that treatment with BB reverses βAR desensitization in RV failure as it does in LV failure.

Here we address the lack of mechanistic studies showing how BB treatment affects βAR responsiveness in RV failure using an experimental rodent model of PAH with RV failure which recapitulates the changes in βAR cascade components seen in human PAH [7]. We investigated the effect of treatment with the β1AR-selective BB metoprolol on βAR responsiveness and expression of key proteins of the βAR signalling pathway and its main regulatory microdomain, the caveola. In LV failure loss of the main caveolar protein, caveolin 3, is causal in aberrant β1 and β2 AR signalling [18], [19].

Metoprolol treatment significantly improved shortening and Ca^2+^ transient response to β1AR stimulation, coinciding with normalisation of the expression and sub-cellular distribution of βAR signalling proteins and caveolar proteins towards that seen in the healthy state. We adapted a computational model of cardiac myocyte β1AR signalling to investigate the impact of observed changes in protein expression and caveolar distribution on function and cAMP signalling. The model predicted changes in compartmentalised cAMP and protein phosphorylation that could explain observed changes in Ca^2+^ cycling in both model and experiment. These data add to work supporting the beneficial use of BB in PAH patients by demonstrating that reverse remodelling of the βAR pathway can occur.

## 2 Methods

### 2.1 Animal model and BB treatment

All work was performed in accordance with the recommendations of the Directive 2010/63/EU of the European Parliament and was approved by the local animal welfare committee at the University of Leeds. Male Wistar rats (200 ± 20 g) received an intra-peritoneal injection of saline (CON) or 60 mg/kg of monocrotaline (MCT) as described previously [20]. MCT induces PAH and RV hypertrophy which progresses to RV heart failure as indicated by signs such as weight loss, lethargy and dyspnoea [20], [21].

Rats were treated with the β1AR-selective blocker metoprolol as described previously [20], [22]. Metoprolol treatment (10 mg/kg/day by voluntary syringe feeding) was started 15 days post MCT injection when PAH and RV hypertrophy were established. This allowed us to examine whether BB treatment initiated after development of PAH could delay or prevent changes in βAR signalling cascade and responsiveness. CON and MCT groups received vehicle solution alone. Experiments were performed when MCT rats displayed signs of heart failure described above or, for CON and MCT + BB rats, at an equivalent time point (23 ± 1 days) [20]. The morphological and functional effects of this BB treatment regime were comprehensively characterised previously.[20], [22].

### 2.2 Isolated RV myocyte response to βAR stimulation

Myocytes were isolated from the RV as described previously [23]. Cell shortening and Ca^2+^ transients were recorded simultaneously in field-stimulated (1 Hz) fura-2-AM loaded RV myocytes using digital edge-detection software (IonOptix, MA, USA) and an OptoScan monochromator (Cairn Research, UK). Cells were continually superfused with Tyrode solution (in mM: 137 NaCl, 5.4 KCl, 0.33 NaH_2_PO_4_, 0.5 MgCl_2_, 5 HEPES, 5.6 glucose, 1 CaCl_2_, pH 7.4) containing β1 or β2 AR selective antagonists. Agonists were applied locally to cells (MPRE8, CellMicro Controls, USA). Bath and local perfusion systems were maintained at 37 °C.

Selective β1AR stimulation was achieved with the βAR agonist isoprenaline (ISO; 1–200 nM) in the presence of the β2AR-selective antagonist ICI 118,551 (ICI; 100 nM). β2AR stimulation was achieved with the β2AR agonist zinterol (ZIN; 0.01–10 μM) in the presence of CGP-20712A (300 nM), a selective β1AR antagonist.

For survival experiments, RV myocytes were superfused with 100 nM ISO (in the presence of ICI). Cells that became hypercontracted or no longer responded to field stimulation when solution was switched back to ICI alone were defined as ‘dead’ cells.

### 2.3 Sucrose density gradient fractionation, Western blotting and RT-PCR

RV homogenate samples were prepared for Western blotting as described previously [23]. RV fractionated samples were prepared as described by [24]. In brief, RV muscle was homogenised for 6 × 20 s in Na_2_CO_3_-based solution (pH 11) and sonicated for 6 × 10 s. After centrifugation at 5000*g* (30 min, 4 °C) for removal of particulate matter, samples were layered on a 45/35/5% discontinuous sucrose gradient. Gradients were centrifuged for 17 h at 280,000*g* (Beckman SW40Ti rotor) at 4 °C. A total of 12 fractions (each 1 mL) were collected following fractionation; fractions 4–12 were used for analysis.

For RV homogenates, all samples were loaded on the same gel and target protein expression was normalised to GAPDH. For RV fractionated samples, equal volumes of pooled (4–5, 9–12) or individual (8) fractions were loaded such that the same amount of total protein was loaded for each heart. Target protein expression is presented as a % of the sum of protein in fractions 4–12.

Extraction and measurement of mRNA was performed as described previously [20]. Transcript expression was normalised to both 18 s and GAPDH housekeeper genes.

### 2.4 Computer simulations

A computational model of canine ventricular myocyte βAR signalling and electrophysiology, developed by (2011), was adapted to incorporate experimentally determined changes in protein expression and caveolar distribution profiles in CON, MCT + BB and MCT. Model parameters were scaled in proportion to protein expression changes measured by Western blotting and sucrose gradient fractionation, relative to CON. Scaling factors and the model parameters used to simulate MCT and MCT + BB groups are provided in Supplementary Table 1. Concentration-dependent inhibition of AC5/6 by Cav 3 was included in the model, based on in vitro findings [25]. Simulations were run for 1000 s (simulation time) without pacing to ensure a steady state, followed by 5 min paced at 1 Hz without β-AR stimulation, then 5 min paced with 100 nmol/L ISO in the presence of β2-AR blockade to replicate a typical experiment.

### 2.5 Statistics

Shortening and Ca^2+^ were measured simultaneously in all cells. Amplitude of cell shortening and kinetics of relaxation in response to βAR stimulation were expressed as % of resting cell length and time from peak shortening to half relaxation (*t*_0.5_) respectively. Ca^2+^ transient amplitude and time from peak Ca^2+^ to half recovery (*t*_0.5_) were expressed as a % change from baseline.

Normality was tested using the Shapiro Wilk test. For protein and mRNA data, where each value represents one biological replicate (animal), CON, MCT and MCT + BB groups were compared using one-way or two-way ANOVA (with the Tukey post-hoc test) if data were normally distributed. For non-normally distributed data, groups were compared using Kruskal Wallis (with the Dunn post-hoc test). Lancaster's mid-P test was used to compare cell survival and the proportion of cells showing a positive inotropic response to selective β1AR or β2AR stimulation between groups. For comparison of survival and inotropic response between groups, α values were corrected for multiple testing using Bonferroni. Cellular contractility and Ca^2+^ fluorescence data were analysed using hierarchical statistics where possible, to account for multiple cells isolated from each heart not being completely independent. The method proposed by Sikkel et al. [26] was employed, which quantifies the intra-class correlation (ICC), representing the degree of data clustering by animal, and adjusts the P threshold accordingly. In most experiments the ICC was <35%, indicating low-to-moderate clustering by animal. Number of cells and rats in each group are reported in figure legends. Ca^2+^ and contractility measurements greater than 2 standard deviations from the mean were automatically excluded as outliers to avoid unphysiological values that can arise due to motion artefacts. Data are shown as mean ± SEM. The threshold for significance was set at P < 0.05. GraphPad Prism 8 and Rstudio were used for all analyses.

## 3 Results

### 3.1 BB treatment improves β1AR responsiveness in the MCT model

**Fig. 1:**
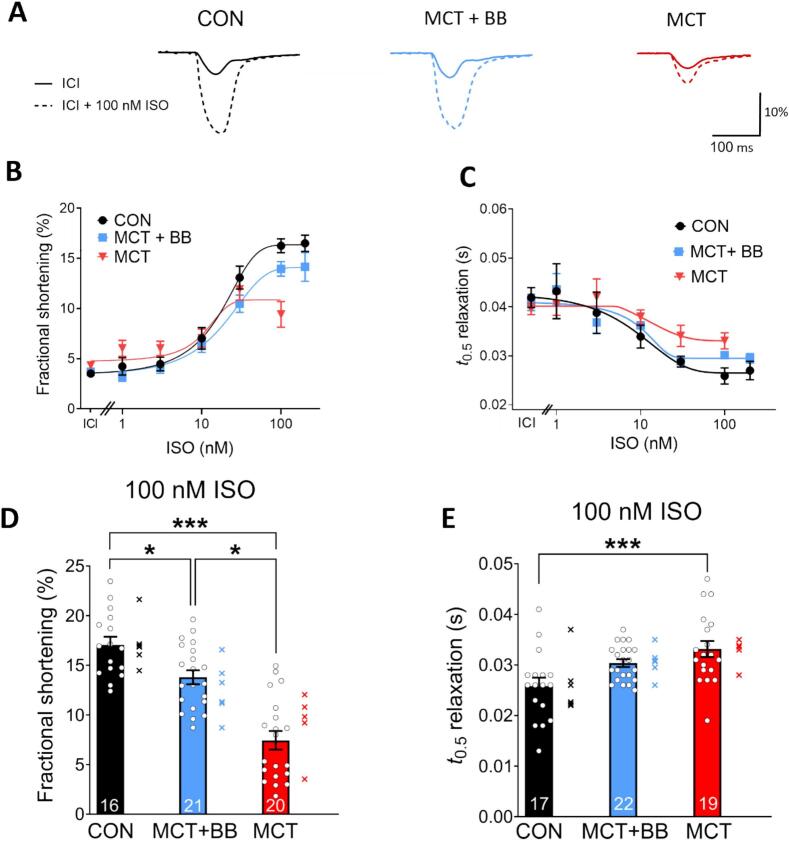
β blocker (BB) treatment in monocrotaline (MCT) animals partially restores the amplitude of RV cardiomyocyte shortening and relaxation kinetics in response to β1AR stimulation. All recordings were made in the presence of the β2AR antagonist ICI 118,551 (ICI). A. Representative traces of cell shortening (as % resting cell length) at baseline (solid line) and following stimulation with 100 nM isoprenaline (ISO; broken line) in control (CON), MCT animals treated with metoprolol (MCT + BB) and the MCT group. B. Log_10_ concentration-response relationship for fractional shortening (as % resting length) with ISO. C. Log_10_ concentration-response relationship for time to half (*t*_0.5_) relaxation with ISO. D. Fractional shortening with 100 nM ISO. E. *t*_0.5_ relaxation with 100 nM ISO. Symbols in B + C represent mean ± SEM of n = 4–66 cells from 5 to 6 rats per group. Open symbols in D + E show individual cell values, bars show mean ± SEM of cell values and cell numbers, and crosses show mean values for each animal. Hierarchical statistics with Bonferroni post hoc correction were performed on D + E to account for multiple cells being isolated from each heart [26]. Number of cells shown on bars, from N = 5–6 animals. 2 CON and 1 MCT + BB cell in panel D, and 1 CON and 1 MCT cell in panel E were automatically excluded as outliers > 2SD from the mean and were not included in analysis. No statistics were performed on B + C. * P < 0.05; *** P < 0.001 between groups indicated.

β1 and β2 AR are the main βAR receptors in the adult myocardium; the β1AR predominates in the healthy heart. We began by assessing the contractile response to selective β1AR stimulation. Representative traces of individual twitches of isolated myocytes from each group are shown in Fig. 1A. In the absence of β1AR stimulation, resting cell length, fractional shortening and the kinetics of relaxation were similar (P > 0.05) in CON, MCT and MCT + BB groups (Fig. S1). CON cells showed robust inotropic (Fig. 1B) and lusitropic (Fig. 1C) responses to β1AR stimulation which were blunted in MCT cells, particularly at high concentrations of ISO (100 nM; P < 0.001; Fig. 1D,E). This difference was partially reversed in MCT animals treated with BB (Fig. 1B,C) as there was greater amplitude of shortening versus cells from untreated MCT rats (Fig. 1D; P < 0.05) and no significant difference in relaxation kinetics between MCT + BB and CON cells in the presence of 100 nM ISO (Fig. 1E; P > 0.05). Thus, in myocytes from the MCT model of RV failure the inotropic and lusitropic responses to β1AR stimulation are reduced, and this is partially recovered with BB treatment.

**Fig. 2:**
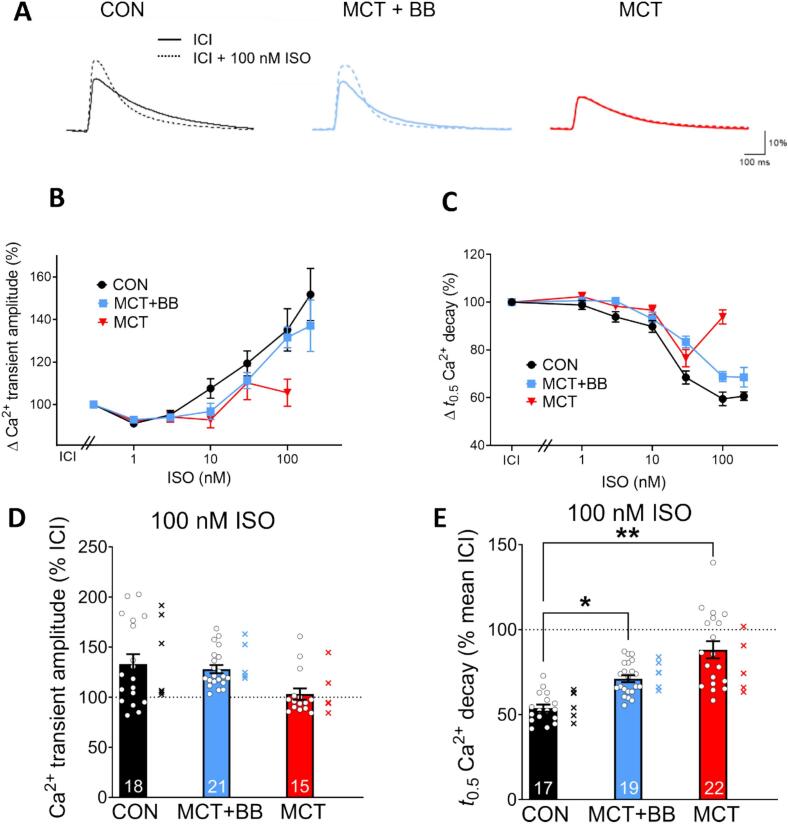
β blocker (BB) treatment in monocrotaline (MCT) animals partially restores the amplitude and kinetics of the Ca^2+^ transient in response to β1AR stimulation. All recordings were made in the presence of the β2AR antagonist ICI 118,551 (ICI). A. Representative traces of individual Ca^2+^ transients (expressed as % change from baseline in fura-2 ratio units, RU) in absence (solid line) and presence (broken line) of 100 nM isoprenaline (ISO) in control (CON), MCT animals treated with metoprolol (MCT + BB) and the MCT group. B. Log_10_ concentration-response relationship for Ca^2+^ transient amplitude response to ISO. The response is expressed as % change from ICI baseline C. Log_10_ concentration-response relationship for change in *t*_0.5_ Ca^2+^ transient decay with ISO. D. Ca^2+^ transient amplitude with 100 nM ISO (expressed as % change from ICI). E. *t*_0.5_ transient decay with 100 nM ISO (expressed as % change from mean ICI). Symbols in B + C represent mean ± SEM of n = 6–27 cells from 5 to 6 rats per group. Open symbols in D + E show individual cell values, bars show mean ± SEM of cell values and number of cells, and crosses show mean values for each animal. Hierarchical statistics with Bonferroni post hoc correction were performed on E to account for multiple cells being isolated from each heart [26]. Data in D were not normally distributed, therefore a Kruskal-Wallis test was performed on the mean values for each animal, which were not significantly different between groups. 1 CON, 1 MCT + BB and 2 MCT cells in panel D, and 1 CON, 1 MCT cell in panel E were automatically excluded as outliers > 2SD from the mean and were not included in analysis. No statistics were performed on B + C. *P < 0.05; **P < 0.01 between groups indicated.

Fig. 2 summarises the Ca^2+^ transient response to β1AR stimulation. Representative traces of individual Ca^2+^ transients in each group are shown in Fig. 2A. In the absence of β1AR stimulation, diastolic [Ca^2+^]_i_ was slightly higher (P < 0.05) in MCT vs. CON and the Ca^2+^ transient amplitude was larger (P < 0.05) in both MCT and MCT + BB compared with CON (Fig. S1). The kinetics of Ca^2+^ transient decay were similar (P > 0.05) in all 3 groups under baseline conditions (Fig. S1F). CON and MCT + BB cells generally showed a robust increase in Ca^2+^ transient amplitude in response to β1AR stimulation with 100 nM ISO, which was attenuated in MCT, however differences between groups were not statistically significant (Fig. 2B). The proportion of cells that responded positively to ISO with an increase in Ca^2+^ transient amplitude was greater in CON (72%) and MCT + BB (100%) compared to MCT (27%) (both P < 0.05 versus MCT; Lancaster's mid-P test with Bonferroni post hoc correction). Similarly, the change in Ca^2+^ transient decay kinetics in response to 100 nM ISO was smaller (P < 0.01) in MCT cells compared to CON (Fig. 2E). Thus, in myocytes from the MCT model of RV failure the responses of Ca^2+^ transient amplitude and decay kinetics to β1AR stimulation were blunted, but could partially recover following BB treatment.

### 3.2 BB treatment improves cell survival in response to β1AR stimulation in the MCT model

Slowed Ca^2+^ transient decay in response to β1AR stimulation in MCT may promote cellular Ca^2+^ overload and cell death. β1AR stimulation with 100 nM ISO (in the presence of ICI 118,551) resulted in some spontaneous contractions, Ca^2+^ waves and cell death. The % of cells that survived 100 nM ISO was significantly different (P < 0.05; Lancaster's mid-P test with Bonferonni post hoc correction) between the 3 groups (n = 50–71 cells from *N* = 7–8 animals). Cell survival was significantly lower (P < 0.01) in the MCT group (52%) compared with CON (80%), but not in MCT + BB group (64%) compared with CON. Thus, BB treatment in MCT animals also protects against myocyte death in response to acute β1AR stimulation.

### 3.3 RV failure and BB treatment have minimal impact on β2AR responsiveness

**Fig. 3:**
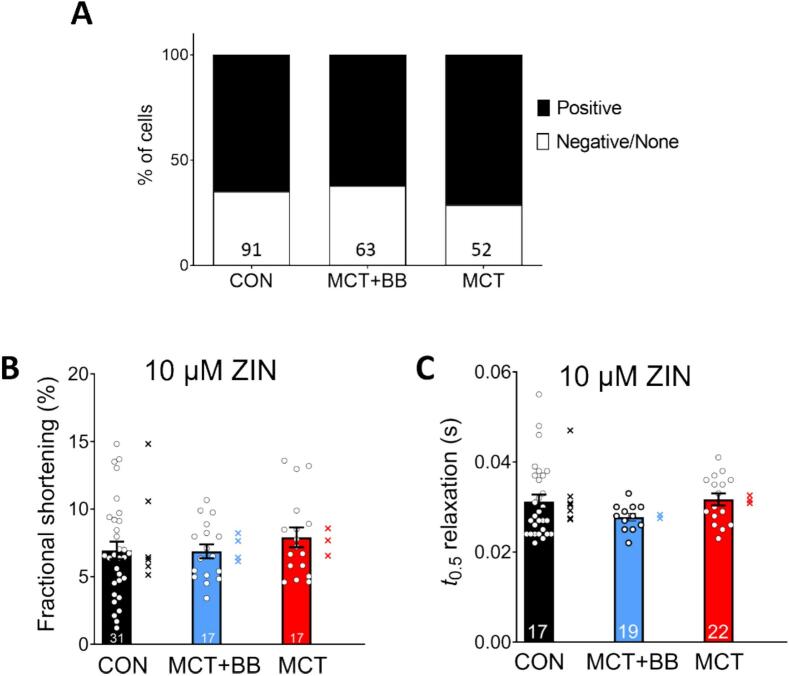
β blocker (BB) treatment does not affect the inotropic and lusitropic response to β_2_AR stimulation in monocrotaline (MCT) animals. All recordings were made in the presence of the β1AR antagonist CGP 20712A. A. The proportion of cells showing a positive inotropic response to zinterol (ZIN; 1–10 μM) was not different in RV myocytes from the 3 groups (*P* > 0.05; Lancaster's mid-P test with Bonferroni post hoc correction; number of cells shown on bars; N = 5–7 animals). B. Fractional shortening (expressed as % resting length) and C. time to half (*t*_0.5_) relaxation with 10 μM ZIN in the presence of CGP. Data are from cells that showed a positive inotropic response to ZIN. Open symbols in B + C show individual cell values, bars show mean ± SEM of cell values and cell numbers, and crosses show mean values for each animal. Hierarchical statistics with Bonferroni post hoc correction were performed on B + C to account for multiple cells being isolated from each heart [26]. Cells were isolated from 2 to 7 rats per group. 2 CON and 1 MCT + BB cell in panel B, and 3 CON, 1 MCT + BB and 1 MCT cell in panel C were automatically excluded as outliers > 2SD from the mean and were not included in analysis. There were no significant differences (P > 0.05) between groups.

Next, we investigated the impact of RV failure and BB treatment on the response to β2AR stimulation with zinterol (ZIN). In healthy myocytes, the inotropic and lusitropic responses to β2AR stimulation are limited by constraints on the amplitude and spread of downstream signalling. Similar to a previous report in healthy ventricular myocytes [24], not all RV myocytes showed a positive inotropic response to β2AR stimulation. The proportion of myocytes that showed a positive inotropic response to ZIN was similar across CON, MCT and MCT + BB groups (P > 0.05; Fig. 3A). In this subset of positive responders, neither fractional shortening nor time to half relaxation in the presence of ZIN were different between the 3 groups (Fig. 3B,C). These data suggest that the beneficial effects of BB treatment in our MCT model of PAH did not primarily involve altered β2AR responsiveness. Given these findings, the next part of the study focused on elements of the β1AR cascade.

### 3.4 BB treatment normalised expression of βAR cascade components in the MCT model

**Fig. 4:**
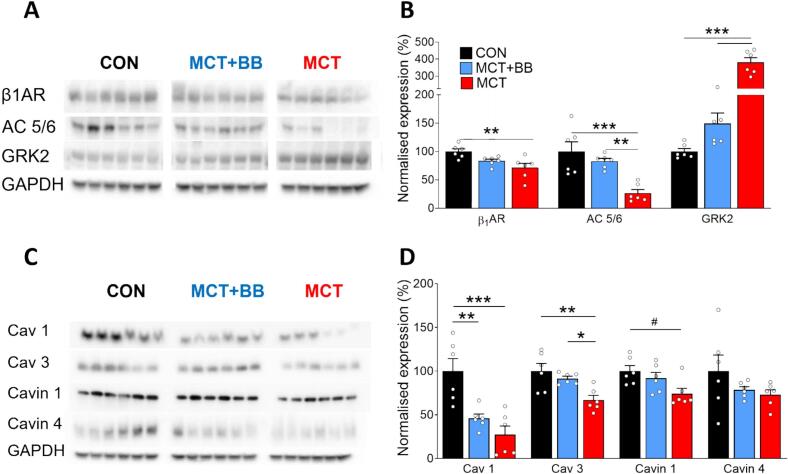
β blocker (BB) treatment normalises total expression of proteins of the β1AR cascade and the caveolar domain in monocrotaline (MCT) animals. A. Western blot from the same gel of β1AR, adenyl cyclase (AC) 5/6 and G protein receptor kinase (GRK) 2 in RV myocardial homogenate. B. Protein expression normalised to GAPDH and expressed as % mean control (CON) value. C. Western blot from the same gel of caveolin (Cav) and cavin in RV myocardial homogenate. D. Expression of caveolar proteins normalised to GAPDH and expressed as % mean control (CON) value. Data in B. and D. are mean + SEM, N = 6 animals in each group. *P < 0.05, **P < 0.01, ***P < 0.001 (ANOVA); # P < 0.05 (Kruskal Wallis).

To understand the mechanisms through which BB treatment restores β1AR responsiveness, we measured the expression of signalling and regulatory proteins of the βAR cascade in the RV myocardium*.* Fig. 4 (A,B) shows expression of β1AR, adenylyl cyclase (AC) 5/6, and G protein receptor kinase 2 (GRK2) in the 3 groups. GRKs phosphorylate the βAR as a trigger for β-arrestin recruitment, its uncoupling from Gs, internalisation, and contribute to desensitization [27]. The GRK2 isoform is responsible for the majority of GRK activity in the rodent heart and is stimulated by sympathetic overdrive [28]. Changes in protein expression in MCT were consistent with reduced β1AR responsiveness in this group: β1AR and AC5/6 expression were reduced whereas GRK2 was markedly increased compared with CON (P < 0.01) (Fig. 4B). Importantly these changes were reversed in MCT + BB, such that there was no longer a significant difference compared with the CON group (P > 0.05). For proteins relevant to the β2AR pathway, β2AR expression was not different (P > 0.05) in the MCT group vs. CON, whereas Gαi3 expression was markedly enhanced in MCT (P < 0.001) and this was reversed by BB treatment (Fig. S2).

Of note, mRNA expression for β1AR, AC5 and AC6 was also reduced in the MCT group (compared with CON), consistent with changes in protein expression, but mRNA levels were not restored by BB treatment (Fig. S3A). GRK2 transcripts were not different between the 3 groups (Fig. S3A). Fig. S3B shows transcripts for additional proteins of the AR cascade, including α1AR, β2AR, Gαi, protein kinase A (PKA) subunits and protein phosphatases (PP). Whilst expression of some transcripts was reduced (α1AR, PKA catalytic subunit α, the protein phosphatase 1 regulatory inhibitor subunit 1A) or increased (Gαi2) in MCT vs. CON (P < 0.01), BB treatment had no impact on expression vs. MCT.

Overall, BB treatment in the MCT model of RV failure reversed changes in protein (but not transcript) expression of key stimulatory and inhibitory components of the β1AR cascade and this could account for the functional recovery of β1AR responsiveness in these animals.

### 3.5 BB treatment normalised expression of caveolar proteins in the MCT model

Caveolins (Cav) and cavins are structural and signalling proteins of caveolae - microdomains that regulate βAR signalling. Cav 1 and cavin 1 are ubiquitously expressed in the myocardium, whereas Cav 3 and cavin 4 are isoforms specific to cardiac myocytes. Loss of Cav 3 has been shown to be responsible for aberrant β1 and β2 AR signalling in animal models of LV failure [18], [19]. Fig. 4(C,D) shows caveolar protein expression. In the MCT group, expression of Cav 1 & 3 and cavin 1 was significantly reduced (P < 0.05), whereas cavin 4 expression was unchanged, compared with CON animals. Expression of Cav 3 and cavin 1 was restored in the MCT + BB group, however Cav 1 expression remained similar (P > 0.05) to MCT levels. There was little correlation between levels of mRNA and protein for the caveolar proteins in the 3 groups (Fig. S4A). Thus, BB treatment can reverse loss of key caveolar proteins in RV failure, and this could contribute to functional improvement by maintaining caveolar signalling domains.

**Fig. 5:**
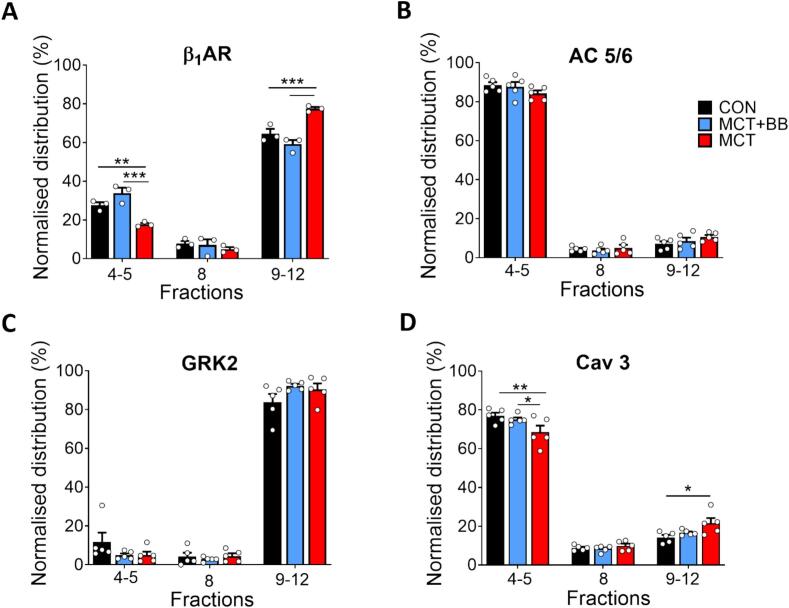
The impact of monocrotaline (MCT) and β blocker (BB) treatment on the membrane and cellular distribution of key elements of the βAR cascade and its regulators. Equal volumes of each pooled (4–5, 9–12) or individual (8) fractions were loaded. Fractions 4–5 contain caveolae; fractions 9–12 contain non-caveolar membranes and cytosolic proteins. The same amount of total protein was loaded for each heart. Distribution is shown as % of the sum of fractions 4–12 for A. β1AR B. AC5/6C. GRK2 and D. Cav3. Data are mean + SEM (N = 6 in each group, apart from A where N = 3). *P < 0.05, **P < 0.01, ***P < 0.001 (two-way ANOVA).

We have shown that MCT animals show marked changes in the global RV expression of both βAR cascade and caveolar microdomain proteins, and that these changes are reversed by BB treatment. However, βAR signalling is exquisitely controlled by the subcellular localisation and scaffolding of proteins. Thus, we looked next at the subcellular distribution of key cascade/caveolar proteins. Myocardial samples were fractionated based on buoyant density which, in turn, depends on cholesterol content. Fractions 4 and 5 contain the cholesterol-enriched caveolar fractions whereas fractions 9–12 contain non-caveolar membranes and cytosolic proteins. Fig. 5 shows the protein distribution between fractions as a % of total protein expression i.e. these represent proportional subcellular distribution between compartments rather than overall protein expression levels.

For the proteins of the βAR cascade, only the β1AR showed significant changes in distribution between groups. β1AR were redistributed from caveolar to non-caveolar fractions/cytosol in the MCT group and this change was reversed by BB treatment (Fig. 5A). Similarly, there was also a small redistribution of Cav 3 from caveolar to non-caveolar fractions in the MCT group which was reversed by BB treatment (Fig. 5D). Very little AC5/6 was detected outside caveolar fractions and this was not different between treatment groups (Fig. 5B). Similarly, no Cav 1 protein was detected outside caveolar fractions in any treatment group (data not shown). We were unable to assess the distribution of cavin 1 or 4 in our fractionated samples, likely due to proteolytic cleavage of PEST motifs within cavins [29]. Together these data suggest that subcellular redistribution of β1AR and βAR- regulatory Cav 3 could contribute to the loss of β1AR responsiveness in PAH and may be reversed by BB treatment.

### 3.6 Computer simulations recapitulate improved β1AR responsiveness

We used computational modelling to assess the impact and relative contribution of protein expression/distribution changes to Ca^2+^ cycling and compartmentalised signalling (cAMP and protein phosphorylation) in MCT and its recovery following BB treatment. The canine cardiac myocyte model of βAR signalling developed by Heijman et al. [30] was modified to incorporate the expression and distribution changes in the βAR signalling pathway components (β1AR, AC5/6, GRK2, Gi) and Cav 3 measured in our experimental groups. These changes are summarised in Supplementary Table 1.

**Fig. 6:**
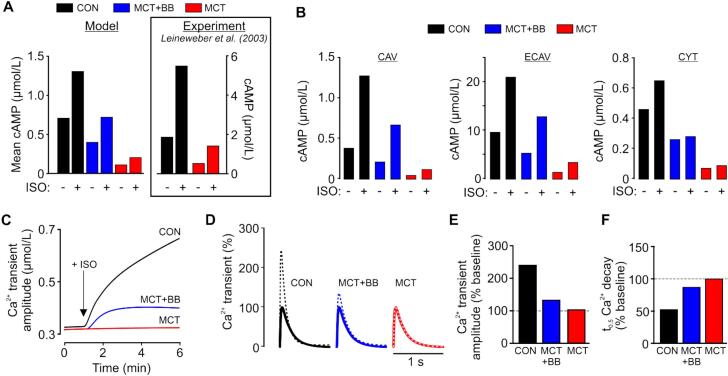
Computer simulation of mean/compartmentalised cAMP and Ca^2+^ transients in response to β1AR stimulation in a cardiac myocyte. Experimental data from control (CON), MCT rats treated with metoprolol (MCT + BB) and the MCT group were used to modify the model of Heijman et al. [30], as outlined in Supplementary Table 1. The response to 100 nM isoprenaline (ISO) was modelled in the presence of β2AR blockade. A. Mean cAMP levels (left panel) under basal conditions and with ISO stimulation. Mean cAMP was predicted based on the relative volume of 3 functional compartments: caveolar (CAV), extra-caveolar (ECAV) and cytosolic (CYT) which are 2, 4 and 67.8% respectively. For comparison, experimental data from Leineweber et al. [31] in rat RV myocyte homogenates is shown (right panel). B. Cyclic AMP levels under basal conditions and with ISO stimulation in individual compartments from the model. C. Time course of Ca^2+^ transient amplitude to ISO. D. Individual Ca^2+^ transients in absence (solid lines) and presence (broken lines) of ISO. E. Ca^2+^ transient amplitude in response to ISO (expressed as % of baseline). F. Ca^2+^ transient decay in response to ISO (expressed as % of baseline).

Fig. 6A (left panel) shows the mean cAMP level in CON, MCT and MCT + BB simulations before and after 5 min ISO stimulation during 1 Hz pacing (in the presence of β2AR blockade). Mean cAMP was reduced by approximately ∼85% in MCT compared to CON under basal conditions (without ISO) and increased ∼2× following ISO stimulation. These model data are in good agreement with experimentally determined cAMP levels in CON and MCT RV myocytes [31] (Fig. 6A; right panel) that showed a ∼ 70% reduction in basal cAMP in MCT compared with CON and ∼ 3× increase in cAMP following ISO stimulation. In our simulated data, BB treatment in MCT partially restored mean cAMP towards CON levels at baseline and in response to ISO (Fig. 6A).

The model comprises 3 functional compartments representing caveolar (CAV), extra-caveolar membrane (ECAV) and cytosolic (CYT) domains. Fig. 6B shows model data for basal and β1AR -stimulated cAMP levels in the 3 compartments. In the CON group, ISO stimulation resulted in a robust increase in cAMP in both CAV and ECAV with only modest increases in the CYT compartment. In all compartments, cAMP levels were substantially reduced in MCT vs CON under basal conditions and with ISO stimulation. Importantly, replicating the changes in protein expression and distribution in MCT + BB partially restored the cAMP response to β1AR stimulation in CAV and ECAV compartments.

Simulated Ca^2+^ transients in response to 1 Hz pacing with β1AR stimulation were in close agreement with experimental data. As shown in Fig. 6(C–E), the robust increase in Ca^2+^ transient amplitude in CON is absent in MCT and partially restored in MCT + BB. Furthermore, ISO increased the rate of Ca^2+^ transient decay (Fig. 6F) in CON but not in MCT, however the response was partially recovered in the MCT + BB group.

### 3.7 Computer simulations predict recovery of protein phosphorylation

**Fig. 7:**
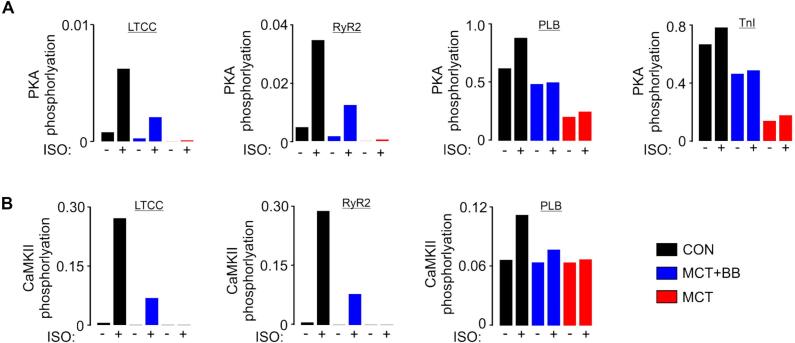
Computational modelling of target protein phosphorylation in response to β1AR stimulation. A. Fraction of protein kinase A (PKA) phosphorylated L-type Ca^2+^ channel (LTCC), ryanodine receptor 2 (RyR2), phospholamban (PLB) and troponin I (TnI) with (+) and without (−) ISO stimulation. B. Fraction of Ca^2+^/calmodulin-dependent kinase II (CaMKII) phosphorylated LTCC, RyR2 and PLB.

Next, we used our computational approach to assess changes in phosphorylation of key regulators of excitation-contraction coupling and targets of PKA and CaMKII following β1AR stimulation, including sarcolemmal (L type Ca channel), sarcoplasmic reticulum (ryanodine receptor, phospholamban) and myofilament (troponin I) proteins. In LV failure, impaired β1AR responsiveness has been ascribed to a marked loss of PKA-dependent signal at the sarcolemma [18]. Fig. 7 shows fractional phosphorylation of PKA (A) and CaMKII (B) targets under basal and ISO-stimulated conditions. In the presence of ISO, the fraction of protein phosphorylated at all sites was lower in MCT vs CON. Of note, BB treatment only recovered a robust ISO response (ISO vs. basal, as seen in CON group) for the L type Ca channel and ryanodine receptor (RyR2). This recovery was seen at both PKA and CaMKII sites.

**Fig. 8:**
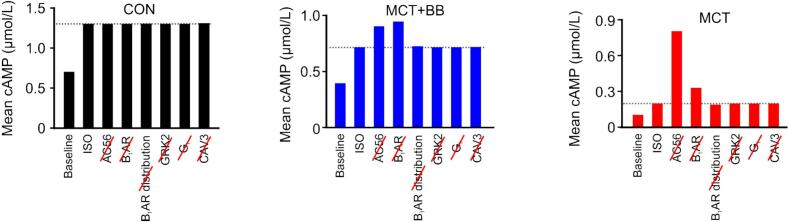
Simulation of the contribution of changes in individual β1AR cascade proteins and Cav 3 to the response to β1AR stimulation. For this simulation, mean cAMP was used as the output measure. Baseline and ISO bars show cAMP in each group with all experimental changes enabled (i.e. the same as Fig. 6A left panel). Additional simulations were performed with each change in protein expression or distribution disabled in turn (indicated by red line) to assess their individual impact on the cAMP response in MCT and MCT + BB groups. For each panel, the dotted line indicates the ISO response with all protein changes enabled. In MCT, cAMP increased most when the disease-associated decrease in expression of AC5/6, and to a lesser extent β1AR, was restored. With BB treatment in the MCT group, AC5/6 and β1AR expression contributed approximately equally to the change in cAMP. (For interpretation of the references to colour in this figure legend, the reader is referred to the web version of this article.)

Finally, we individually restored protein expression and distribution parameters in MCT and MCT + BB groups to the equivalent CON values to determine their relative contribution to β1AR responsiveness. Mean cAMP levels were used as the output measure (Fig. 8). Loss of AC5/6 expression was the principal determinant of the impaired β1AR responsiveness in MCT; there was also a minor contribution from the loss of β1AR. By contrast, both AC5/6 and β1AR expression accounted to a similar extent for the change in β1AR responsiveness in the MCT + BB group. The large increase in GRK2 expression in MCT vs CON had minimal impact on β1AR responsiveness, likely due to the slow rate of receptor desensitization by GRK2 which is not captured in the timescale of these simulations [30]. Cav 3 inhibits AC5/6 in vitro [25] and therefore reduced Cav 3 expression in MCT might compensate for reduced β1AR responsiveness, however our simulated data suggests this effect was very small. Simulations did indicate that altered β1AR distribution in MCT may counteract reduced β1AR responsiveness to a small degree.

Overall, these simulations indicate that much of the improvement in β1AR responsiveness in RV failure treated with BB can be attributed to increased cAMP production in CAV and ECAV compartments due to recovery of AC5/6 and β1AR expression.

## 4 Discussion

Current therapy for RV failure in PAH targets the pulmonary circulation, but there is a movement towards treatment of the failing RV directly [32]. BB treatment addresses the maladaptive consequences of increased sympathetic drive seen in RV failure [33]. Previous work in the MCT model of PAH showed that BB treatment enhanced cardiac function, increased SERCA expression, and improved survival, as it does in heart failure arising from other causes [10], [17], [20]. Here we show, using RV myocytes from the MCT model, that BB treatment also recovers β1AR responsiveness. Our experimental and modelling data indicate how altered expression and distribution of key elements of the βAR signalling cascade and its regulators contribute to this.

### 4.1 Choice of BB

BB used to treat heart failure in the clinic include 2nd generation drugs such as metoprolol (β1AR selective) as well as 3rd generation drugs such as carvedilol which are βAR non-selective but have additional vasodilatory effects [16]. Metoprolol and carvedilol each confer particular benefits in the treatment of PAH: carvedilol because it targets the pulmonary vasculature as well as the heart [34]; metoprolol because it is a complete inverse agonist which holds the βAR1 in a constitutively inactive state preventing its phosphorylation and internalisation, so maintaining βAR responsiveness [50]. Recently diagnosed PAH patients receiving β1AR selective blockers for existing co-morbidities, such as arrhythmias, coronary artery disease, and hypertension had better outcomes than those treated with non-selective BB [15]. Here we used metoprolol to understand the impact of selectively targeting the myocardial β1AR while minimizing confounding effects on the pulmonary vasculature.

### 4.2 BB treatment restores β1AR responsiveness

RV myocytes from the MCT rat showed blunted inotropic and lusitropic responses to β1AR stimulation, consistent with parallel changes in Ca^2+^ transient amplitude and kinetics. These changes were reversed by BB treatment. In tandem, there was reduced myocardial expression of β1AR and AC5/6 and increased expression of GRK2 in the MCT group and, again, these changes were reversed by BB treatment. Altered protein expression could explain blunted inotropic and lusitropic responses to β1AR stimulation in RV failure and its recovery with BB treatment.

Increased plasma and tissue levels of epinephrine and norepinephrine have been reported in MCT rat hearts [31], [35]. Increased sympathetic activation in vivo supports RV contraction against greater afterload in PAH, but coincides with a decrease in catecholamine-mediated cAMP production, with the effect being more pronounced in the RV compared to the LV [31], [35], [36]. Acutely isolated cells are devoid of intrinsic sympathetic activation, thus allowing assessment of their function in the absence or presence of selective β1/β2-AR stimulation. The changes in protein expression that we find in MCT are in line with other reports in this model [31], [35], [36]. Importantly, they also recapitulate the remodelling of the βAR cascade seen in human PAH: down-regulation of β1AR and blunted baseline and stimulated adenylyl cyclase activity [37]. Although BB treatment has been shown to remodel the β1AR pathway in LV failure, this is the first report of its reversal of cardiac β1AR responsiveness, at both functional and protein level, in RV failure. We cannot exclude the possibility that some of the beneficial outcomes of BB treatment in PAH may also include indirect effects acting via, e.g. altered loading of the LV, reduction in heart rate and overall energy demand, or increase in coronary perfusion [22]. This does accord with limited information available regarding the impact of BB treatment in PAH patients. For example, in the PAH Treatment with Carvedilol for Heart failure (PAHTCH) trial, carvedilol recovered leucocyte βAR density and enhanced urinary cAMP excretion - a surrogate of AC activity [3].

Previous work in this model of RV failure has shown a pro-arrhythmic phenotype and negative contraction-frequency relationship which is partially recovered by BB treatment [20], [21]. These data can be considered alongside our observations here of impaired functional responses and myocyte survival with β1AR stimulation in MCT which are recovered with BB treatment. Together they describe a situation where the failure of the RV to respond to increased demand can be improved by BB treatment.

### 4.3 BB treatment restores caveolar protein expression

Caveolae, small invaginated lipid rafts lined with Cav and cavin proteins, are crucial for the organisation and modulation of the β AR signalling cascade in the cardiac myocyte [38]. In the MCT model of RV failure, there was down-regulation of caveolar proteins (Cav 1, Cav 3 and cavin 1) in association with some re-distribution of Cav 3 away from caveolar fractions. Taken together, in the context of current knowledge of the dependence of caveolae on Cav and cavin proteins [39], [40], our data suggest that there is a loss of structure and organisation of the cardiac caveolar microdomain in the failing RV. Our data suggest that recovery of caveolar proteins following BB treatment could explain some of the benefits of BB in RV failure.

Loss of caveolar patency in MCT and its recovery with BB could contribute to changes in the inotropic response to β1AR stimulation. For example, in models of LV failure, there are reports of caveolar disruption, indexed by reduced Cav 3 expression, loss of Cav 3 membrane localisation and fewer caveolae [18], [41], [42]. Using tethered A-kinase activity reporter proteins (AKARs), Barbagallo et al. [18] observed decreased β1AR signals at the sarcolemma in LV failure which was corrected by Cav 3 overexpression in failing myocytes, suggesting Cav 3 dependency. Overexpression of Cav 3 in LV failure restored t-tubular localisation of the L type Ca^2+^ channel necessary for its effective coupling with RyR2 [43]. The L-type Ca^2+^ current makes a major contribution to increased Ca^2+^ transient amplitude (and therefore inotropy) following β1AR stimulation in the healthy myocyte [44]. Thus, experimental evidence supports a role for Cav 3-dependent changes in L-type Ca^2+^ current in the blunted β1AR inotropic response observed in RV failure, and its reversal with BB treatment.

By contrast, the blunted lusitropic responses to β1AR stimulation (in conjunction with slowed Ca^2+^ transient decay) observed in MCT, and their recovery with BB treatment are most likely to be due to changes in signals at the sarcoplasmic reticulum (i.e. phospholamban). We previously found impaired diastolic function in the MCT model of PAH [23], and interestingly a small interventional trial found that acute administration of short acting B1AR selective blocker esmolol improved diastolic function during exercise in PAH patients [45]. In LV failure, only minimal (non-significant) changes in sarcoplasmic reticulum AKAR signals and phospholamban phosphorylation occurred and the former was not reversed by correcting Cav 3 expression in failing myocytes [18]. Although we did not measure substrate phosphorylation in our study, by incorporating expression changes in β1AR cascade/caveolar protein into a cardiac myocyte computational model we could interrogate its possible changes in RV failure and the impact of BB treatment (see Section 4.4).

In terms of β2AR, loss of caveolae disruption with cholesterol scavenging, or as seen in LV failure, removes the inhibitory influences that normally prevent access of cAMP signal to the SR and myofilaments [18], [24]. Given the evidence of caveolar disruption in MCT, we expected that there might be an increase in proportion of MCT cells that responded positively to selective β2 stimulation, or with greater inotropic or lusitropic response, however we did not find conclusive evidence of this. There are many reasons why this may be; the simplest of which is the degree of disassembly of caveolae. Although Western blotting is semi-quantitative, comparison of changes in Cav3 expression in LV vs. RV failure suggest this may contribute. Much greater reduction in expression of Cav3 by ∼75% was associated with myofilament propagation of β2AR signal in LV [18], whereas this was around two-fold greater protein loss than in our MCT model.

### 4.4 What is responsible for blunted β1AR responsiveness in RV failure?

Our experimental data show blunted β1AR responsiveness in MCT, rescued by BB treatment. In the context of current literature, the effects of BB treatment in MCT could be explained by both changes in β1AR cascade protein expression/distribution and caveolar protein expression/distribution. Next, we attempted to dissect out the functional relevance of changes in protein expression and caveolar domain organisation.

Computational modelling is an ideal tool to use in this situation as it permits simulation of compartmentalised signalling (cAMP, substrate phosphorylation) and allowed us to predict the relative contribution of changes in individual protein expression to myocyte function. Here we used an established βAR signalling model [30], and incorporated experimentally observed changes in protein expression and distribution in MCT and MCT + BB groups. The model allowed us to predict underlying changes in compartmentalised cAMP in our 3 groups which is challenging to measure experimentally. The relative magnitude of differences between model predicted mean cAMP levels was consistent with experimental data (for CON and MCT groups) reported by others [31]. The principal increase in cAMP in control simulations was in the CAV and ECAV compartments which fits with the distribution of the β1AR between caveolar and extra-caveolar sarcolemma shown here and by others [46], [47], [48]. BB recovered depressed cAMP signals in CAV and ECAV compartments. The simulated functional output (Ca^2+^ transient) response to β1-AR stimulation was similar to experimental data, providing validation of the modelling approach.

The model also allowed us to predict the impact of MCT and BB treatment on phosphorylation of key proteins that underlie inotropic and lusitropic responses to β1AR. BB treatment was predicted to recover β1AR-stimulated phosphorylation of determinants of Ca^2+^ transient amplitude (LTCC and RyR2), at both PKA and CaMKII sites. By contrast, there was only a small return of β1AR-stimulated phosphorylation of proteins that determine myocyte lusitropy (PLB and TnI), although BB treatment did partially reverse low basal levels of phosphorylation at these sites.

The computational modelling suggests that the reduction in expression of AC5/6 makes the major contribution towards blunted β1AR responsiveness in MCT and its recovery with BB treatment. The sensitivity of the sucrose gradient technique was such that we were unable to experimentally detect a significant redistribution of proteins that are very highly localised in the caveolar domain (AC5/6 and Cav 1) in the MCT group, although such redistribution is expected to exist. Consequently, we did not model distribution changes in AC5/6 or Cav1. Cav3 places a brake on AC5/6 activity [25], and the greater availability of Cav3 in MCT + BB vs. MCT could act to blunt AC5/6 activity, contributing to impaired β1AR responsiveness with BB treatment despite apparent recovery of AC5/6 expression in this group. Indeed, when Cav3 inhibition of AC5/6 was incorporated into the model it did slightly suppress the rise in cAMP in control that was lessened in MCT, however the overall magnitude of direct inhibition was small.

### 4.5 Limitations

No animal model exactly recapitulates all the features of human disease. However, the changes in the RV of MCT-treated rats display all the key characteristics of myocardial remodelling associated with increased sympathetic drive (i.e. as seen in human LV and RV failure). The computational model employed here uses canine ventricular electrophysiology, which is similar to human but has important differences in shape and duration compared to the rodent action potential. As such, we did not attempt to reproduce failing electrophysiology and instead focus on the consequences of changes in βAR signalling protein expression and distribution. We have previously characterised changes in electrophysiology and Ca^2+^ handing in the MCT model, including action potential lengthening due to reduced potassium channel and subunit expression [21] and diastolic sarcoplasmic reticulum Ca^2+^ leak due to reduced SERCA2a and PLB expression and phosphorylation [20]. The approximately 8-day BB treatment was chosen to compare β-AR signalling pathway and responsiveness with untreated time-matched MCT rats, which is a relatively short treatment period. We previously showed that the median survival time increased from 23 days in MCT rats to 31 days in MCT + BB rats, but do not currently know what happens to β-AR signalling at this more advanced stage with longer treatment.

### 4.6 Conclusion

PAH is a relatively rare disease but carries a poor prognosis. β-blockers are not currently recommended treatments for PAH [11], however emerging clinical and experimental evidence suggests they may be tolerated or provide some benefits in select groups of PAH patients with comorbidities, such as arrhythmias. More work is needed to understand which groups are more likely to respond positively and possible mechanisms involved. Here we show BB treatment resulted in functional re-sensitisation of β1AR responses which can be explained by recovery of signalling protein expression in a preclinical model. Although we see evidence of caveolar disruption in MCT which is corrected by BB treatment, our modelling data suggest that reversal of the marked reduction in AC5/6 expression plays the key role in recovery of responsiveness. As right heart function determines quality of life, exercise capacity and mortality [49], understanding how therapies modulate signalling pathways could provide an evidential basis for identifying groups of PAH patients that may benefit from alternative treatment approaches.

## CRediT authorship contribution statement

**Ruth Norman:** Writing – review & editing, Writing – original draft, Visualization, Investigation, Formal analysis. **Rachel Stones:** Writing – review & editing, Writing – original draft, Investigation. **Amelia S. Power:** Writing – review & editing, Writing – original draft, Investigation. **Ed White:** Writing – review & editing, Writing – original draft, Supervision, Resources, Project administration, Funding acquisition, Conceptualization. **Sarah C. Calaghan:** Writing – review & editing, Writing – original draft, Supervision, Resources, Project administration, Funding acquisition, Conceptualization. **Ewan D. Fowler:** Writing – review & editing, Writing – original draft, Visualization, Methodology, Investigation, Formal analysis.

## Funding

This work was supported by the 10.13039/501100000777University of Leeds, UK (scholarship to RN) and by the 10.13039/501100000274British Heart Foundation (grant numbers FS-IBSRF-21-25071 to EDF, PG/17/84/33372 to SC and PG/13/3/29924 to EW).

## Declaration of competing interest

The authors declare the following financial interests/personal relationships which may be considered as potential competing interests:

Ewan Fowler reports financial support was provided by British Heart Foundation. If there are other authors, they declare that they have no known competing financial interests or personal relationships that could have appeared to influence the work reported in this paper.
