# Supplementary material for: The β1 adrenoceptor (AR) blocker metoprolol normalises βAR cascade and caveolar protein expression and restores β1AR responsiveness in failing right ventricular myocytes
Source: J Mol Cell Cardiol Plus. 2026 Jul 17;17:100857. doi: 10.1016/j.jmccpl.2026.100857 (PMC13393817; doi:10.1016/j.jmccpl.2026.100857)
Supplement: Supplementary file 1 — Supplementary material 1 [file mmc1.pdf]

Supplementary Material

1 Supplementary methods

1.1 Computer simulations

A computer model of cardiac myocyte β-adrenergic signalling (Heijman et al., 2011) that was based on a model of canine ventricular myocyte electrophysiology and Ca^{2+} handling (Decker et al., 2009) was modified to interrogate changes in expression and distribution of signalling cascade proteins in our experimental groups. The model considers compartmentation of signalling by assigning signal components to 3 compartments: caveolar (CAV), extra-caveolar membrane (ECAV) and cytosolic (CYT). Table S1 shows scaling factors used to adjust model parameters based on our own experimental findings and published literature.

β1-AR distribution between CAV, ECAV and CYT compartments was set according to sucrose gradient fractionation data, with CAV corresponding to fractions 4/5. In healthy pig ventricle 80% of β1AR was membrane localised, whereas in failing ventricle this fell to 65%, with the remaining β1AR cycling through early/late endosomes (Perrino et al., 2005). These approximate distributions were achieved by setting β1AR in CAV domain in the model to the fraction 4/5 distribution measured by sucrose gradient fractionation (Fig. 5A) and leaving the proportion of β1-AR in the ECAV domain unchanged (0.487).

Direct inhibition of AC5/6-mediated cAMP production by Cav 3 in the CAV domain was incorporated into the model, assuming ~13% inhibition per µmol/L Cav 3, as reported *in vitro* by Toya *et al.* (1998). The precise concentration of Cav 3 in caveolae is not known so the concentration in CON group was set to an approximate mean cellular concentration of Cav3 (1 µmol/L). Cav 3 in each group was scaled according to the relative expression and distribution of Cav3 in fraction 4/5 (Fig. 4D & Fig 5D).

2 Supplementary Figures and Tables

2.1 Supplementary Figures

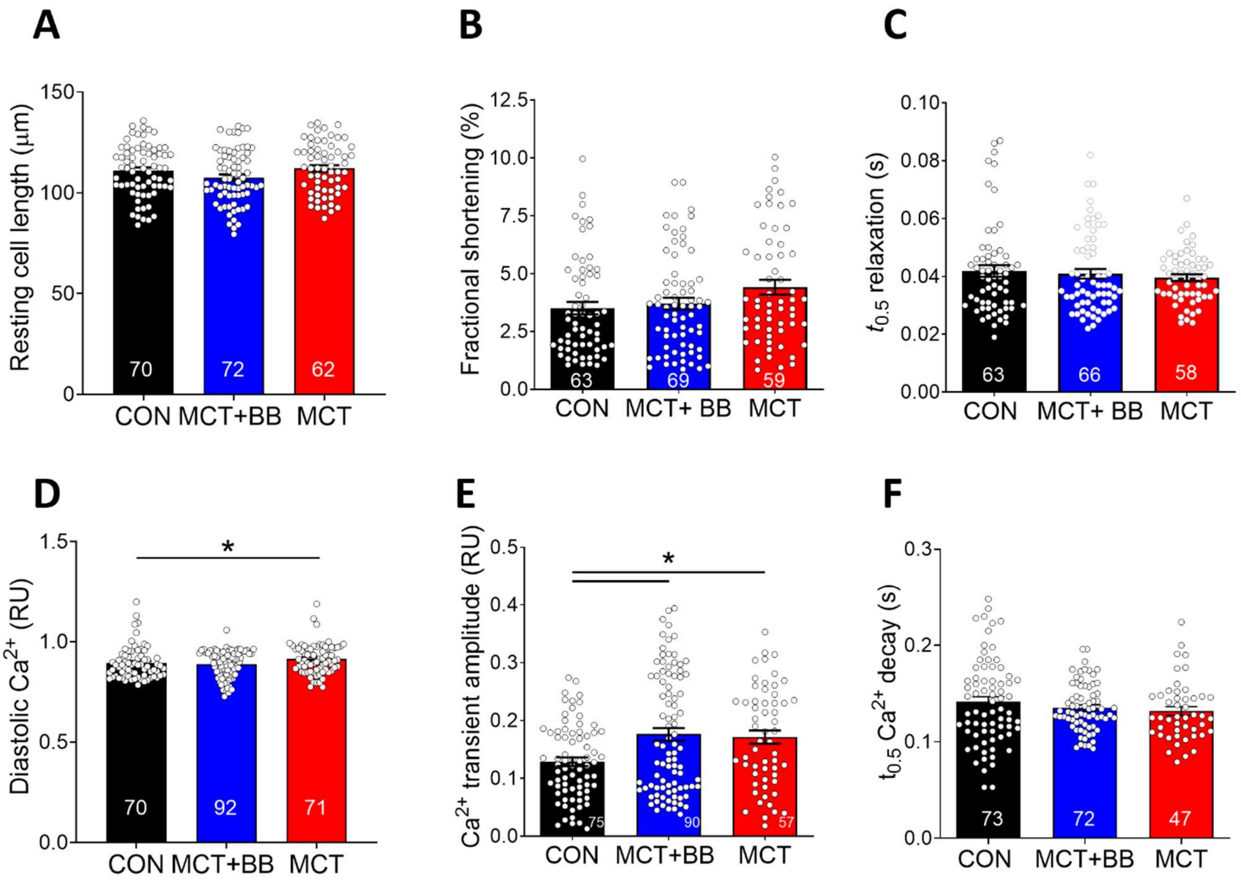

Supplementary Figure 1. Contractile and $[Ca^{2+}]_i$ handling parameters measured under basal conditions in RV myocytes isolated from control (CON), monocrotaline (MCT) and MCT-βAR blocker (MCT+BB) animals. Parameters were measured in the absence of βAR stimulation (with ICI 118,551) **A** Resting cell length, **B** fractional shortening (expressed as a % of resting cell length) and **C** time to half ($t_{0.5}$) relaxation **D** Diastolic $[Ca^{2+}]_i$ and **E** Ca^{2+} transient amplitude expressed as fura 2 ratio units (RU) and **F** time to half ($t_{0.5}$) decay. Data are mean + SEM, number of cells shown on bars (N=5-7 animals). *P<0.05 (Kruskal Wallis).

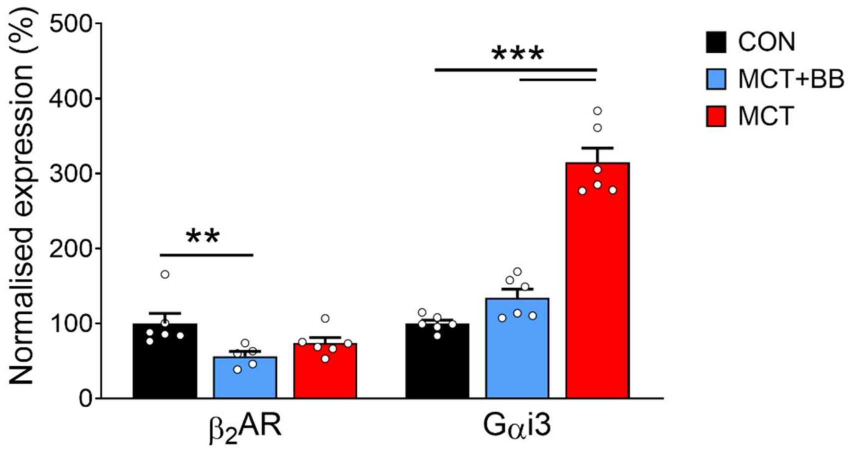

Supplementary Figure 2. β blocker (BB) treatment normalised protein expression of Gαi3 but not the β2AR, in monocrotaline (MCT) animals. Protein expression (normalised to GAPDH) and expressed as % mean CON value. Data are presented as mean + SEM (N=6). **P<0.01, ***P<0.001 (ANOVA).

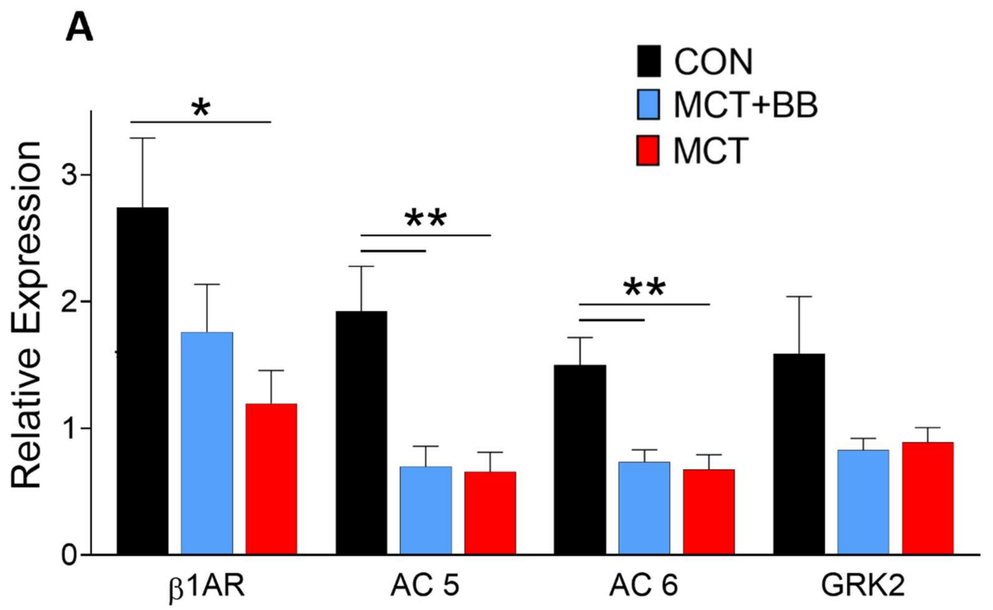

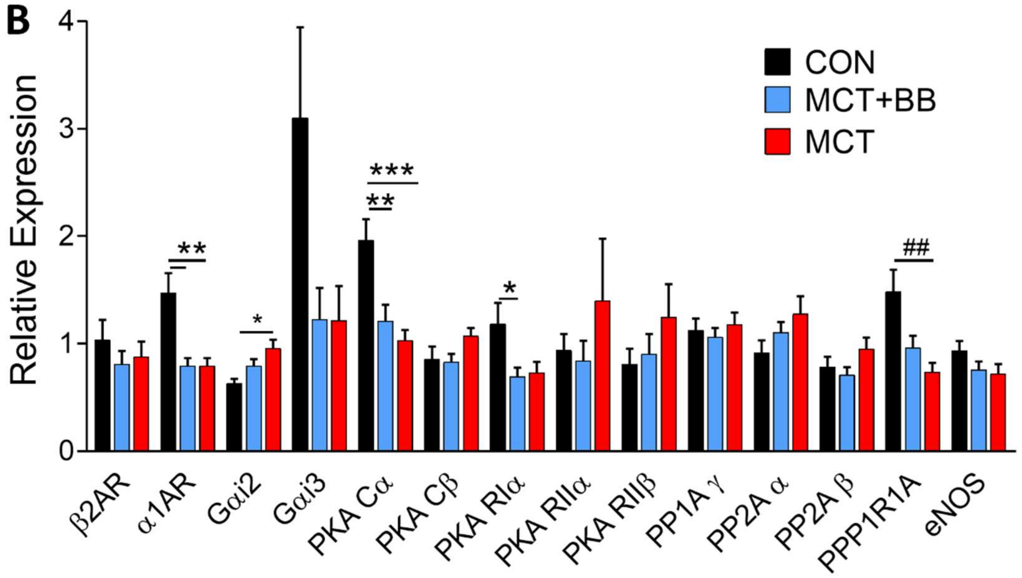

Supplementary Figure 3. β blocker (BB) treatment failed to normalise mRNA expression for elements of the AR signalling cascade in monocrotaline (MCT) animals. **A** Transcripts relevant to β1AR for which protein expression levels are shown in Figure 4A. **B** Miscellaneous transcripts for elements of the adrenoceptor signalling cascades. All mRNA levels are normalised to GAPDH and 18S. AC, adenylyl cyclase; GRK, G-protein receptor kinase; AR, adrenoceptor; PKA C, catalytic subunit of protein kinase A; PKA R, regulatory subunit of PKA; PP, phosphatase; PPP1R1A protein phosphatase 1 regulatory inhibitor subunit 1A; eNOS, endothelial nitric oxide synthase. Data are presented as mean + SEM; N=10 animals *P<0.05, **P<0.01. ***P<0.001 (ANOVA); ## P<0.01 (Kruskal Wallis).

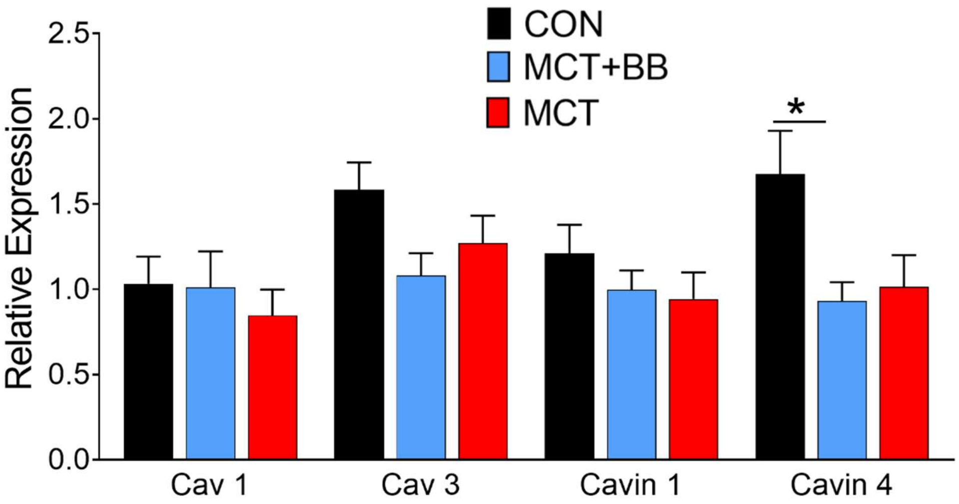

Supplementary Figure 4. The effect of β blocker (BB) treatment on transcript expression of key caveolar components in monocrotaline (MCT) animals. Expression normalised to GAPDH and 18S. Cav, caveolin. Data are presented as mean + SEM (N=10). * P<0.05, (ANOVA).

2.2 Supplementary tables

Table S1: Values used for adjustments to Heijman *et al.* (2011) model based on experimental finding in this study and published literature. [a]Cav 3 scaffolding peptide dose-dependently inhibits adenylyl cyclase 5 (Toya *et al.* 1998) [b]Assuming ~1 µmol/L Cav 3 in normal cardiac myocytes

Parameter	Variable	Value/Scaling factor			Rationale
		CON	MCT+BB	MCT	
Total β1AR protein	R_b1_tot	1	0.8	0.7	Experimental findings (relative to CON)
β1AR subcellular distribution in caveolar domain	f_Rb1_CAV	0.28	0.34	0.18	Experimental findings
Total AC5/6 protein	AC56_tot	1	0.8	0.25	Experimental findings (relative to CON)
Amplification factor for AC56 in CAV domain	AF56_CAV	37.24	37.65	38.91	Scale AC56 activity depending on Cav3[a]
Total Cav3 protein	cav3_tot	1	0.9	0.66	Experimental findings (relative to CON)[b]
Cav3 subcellular distribution in caveolar domain	f_CAV3_CAV	0.76	0.76	0.68	Experimental findings
Total Gi protein	Gi_tot	1	1.3	3.1	Experimental findings (relative to CON)
Total GRK2 protein	GRK2	1	1.5	4	Experimental findings (relative to CON)

Blots underlying Western blot data.

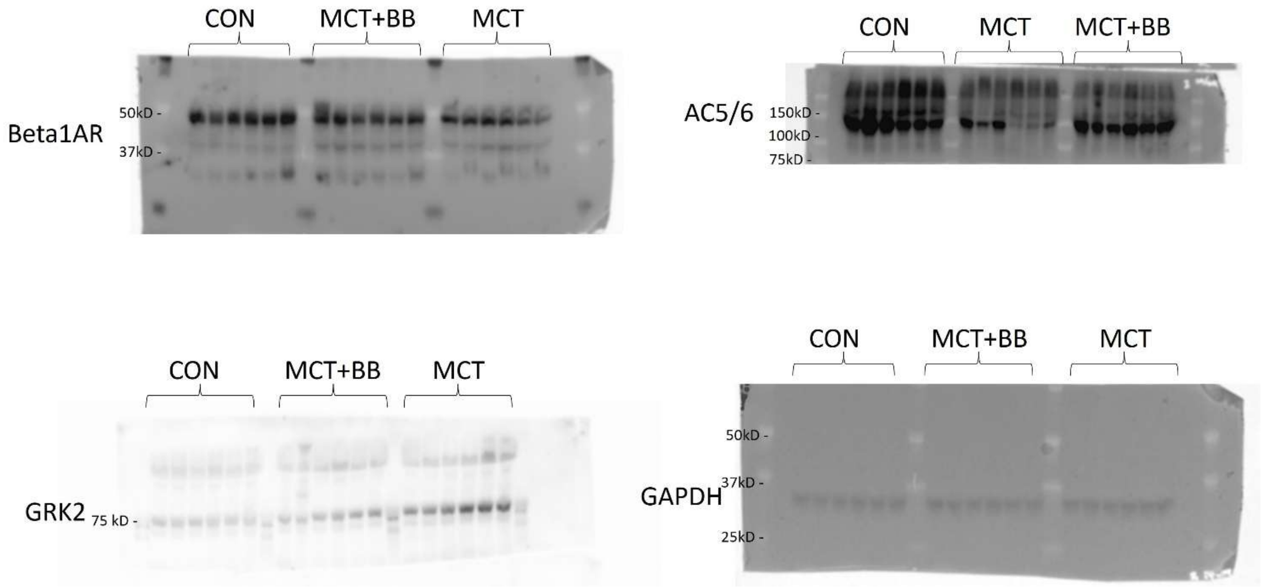

Blots underlying data in Fig4A showing molecular weight markers.

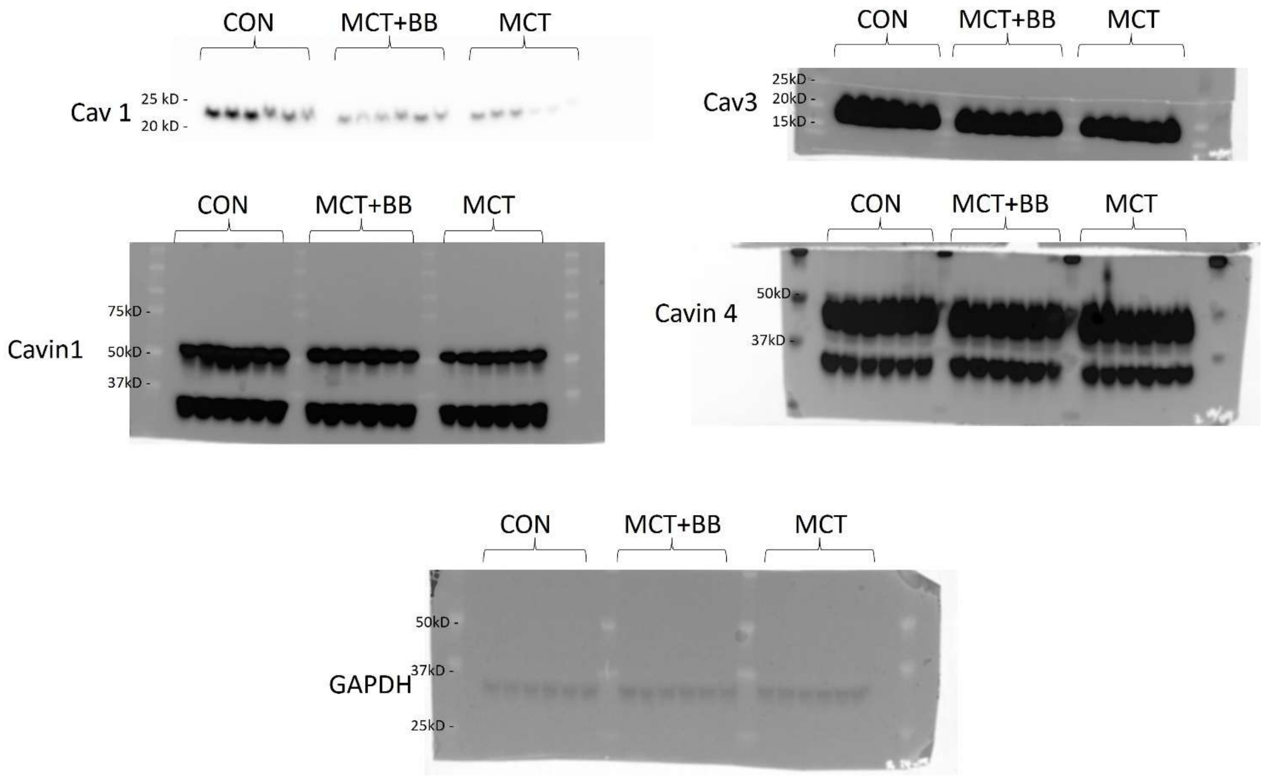

Blots underlying data in Fig4C showing molecular weight markers.

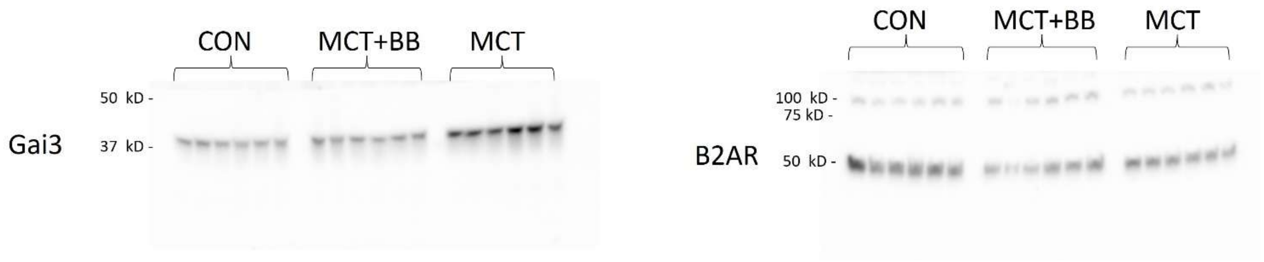

Blots underlying data in Supplementary Figure 2 showing molecular weight markers.

3 Supplementary references

Decker, K. F., Heijman, J., Silva, J. R., Hund, T. J., and Rudy, Y. (2009). Properties and ionic mechanisms of action potential adaptation, restitution, and accommodation in canine epicardium. *Am J Physiol Heart Circ Physiol* 296, H1017-1026. doi: 10.1152/ajpheart.01216.2008

Heijman, J., Volders, P. G. A., Westra, R. L., and Rudy, Y. (2011). Local control of β-adrenergic stimulation: Effects on ventricular myocyte electrophysiology and Ca2+-transient. *J Mol Cell Cardiol* 50, 863–871. doi: 10.1016/j.yjmcc.2011.02.007

Perrino, C., Naga Prasad, S. V., Schroder, J. N., Hata, J. A., Milano, C., and Rockman, H. A. (2005). Restoration of β-Adrenergic Receptor Signaling and Contractile Function in Heart Failure by Disruption of the βARK1/Phosphoinositide 3-Kinase Complex. *Circulation* 111, 2579–2587. doi: 10.1161/CIRCULATIONAHA.104.508796

Toya, Y., Schwencke, C., Couet, J., Lisanti, M. P., and Ishikawa, Y. (1998). Inhibition of adenylyl cyclase by caveolin peptides. *Endocrinology* 139, 2025–31. doi: 10.1210/endo.139.4.5957
